# Canonical (CD74/CD44) and Non-Canonical (CXCR2, 4 and 7) MIF Receptors Are Differentially Expressed in Rheumatoid Arthritis Patients Evaluated by DAS28-ESR

**DOI:** 10.3390/jcm11010120

**Published:** 2021-12-27

**Authors:** Gabriela Athziri Sánchez-Zuno, Richard Bucala, Jorge Hernández-Bello, Ilce Valeria Román-Fernández, Mariel García-Chagollán, Ferdinando Nicoletti, Mónica Guadalupe Matuz-Flores, Samuel García-Arellano, Judith Alejandra Esparza-Michel, Sergio Cerpa-Cruz, Edsaúl Emilio Pérez-Guerrero, José Francisco Muñoz-Valle

**Affiliations:** 1 Instituto de Investigación en Ciencias Biomédicas, Centro Universitario de Ciencias de la Salud, Universidad de Guadalajara, Jalisco 44340, Mexico; athziri.sanchez@alumnos.udg.mx (G.A.S.-Z.); jorge89_5@hotmail.com (J.H.-B.); valerofedz@gmail.com (I.V.R.-F.); maye_999@hotmail.com (M.G.-C.); matuzmonica@gmail.com (M.G.M.-F.); sm_0490@hotmail.com (S.G.-A.); judith.ale@hotmail.com (J.A.E.-M.); edsaul.perezg@academicos.udg.mx (E.E.P.-G.); 2 Department of Medicine, Section of Rheumatology, Yale University School of Medicine, New Haven, CT 06520, USA; richard.bucala@yale.edu; 3 Department of Biomedical and Biotechnological Sciences, University of Catania, 95123 Catania, Italy; ferdinic@unict.it; 4 Servicio de Reumatología, O.P.D. Hospital Civil de Guadalajara “Fray Antonio Alcalde”, Jalisco 44280, Mexico; sacer04@prodigy.net.mx

**Keywords:** rheumatoid arthritis, flow cytometry, MIF, inflammation, DAS28

## Abstract

Macrophage migration inhibitory factor (MIF) significantly contributes to rheumatoid arthritis (RA) pathogenesis. We aimed to evaluate the canonical (CD74/CD44) and non-canonical MIF receptors (CXCR2,4 and 7) expression and sCD74 to establish their association with RA clinical activity according to DAS28-ESR. Methodology: 101 RA patients with different clinical activities (remission (*n* = 27), low (*n* = 16), moderate (*n* = 35) and high (*n* = 23)) and 9 control subjects (CS) were included. Expression was evaluated by flow cytometry and levels of soluble CD74 (sCD74) by ELISA. Data analysis was performed with FlowJov10.0, STATAv12.0, and GraphPad Prism v7.0. Results: According to disease activity, CXCR7 expression (percentage of expression and mean fluorescence intensity (MFI)) was higher in granulocytes from patients in remission, while the expression of CXCR4 was higher in patients with high disease activity (*p* < 0.05). The expression of CD74 was higher in B cells (*p* < 0.05) and monocytes (*p* < 0.01) from patients in remission. Regarding sCD74 levels these were higher in patients with high disease activity when compared to those in remission (*p* <0.05). Conclusions: The results support the need for further study of the role of sCD74 as a soluble MIF decoy receptor, sequestering it to negatively regulate MIF signaling though its membrane receptors. The expression patterns of CXCR4 and CXCR7 show that the latter is a scavenger-type receptor that prevents endocytosis and even degradation of CXCR4 under inflammatory conditions.

## 1. Introduction

Rheumatoid arthritis (RA) is a progressive autoimmune disease of unknown etiology that represents the most prevalent inflammatory autoimmune disease worldwide (1%) [1]. This disease is characterized by inflammation of the synovial membrane (synovitis), which is mainly driven by a leukocyte infiltrate in the synovium, as well as positive feedback cycles of inflammatory stimuli. The accumulation of joint damage can trigger cartilage destruction and bone resorption that will ultimately lead to irreversible disability [2,3]. The most widely used and validated score to assess clinical activity in RA patients is the Disease Activity Score 28 incorporating erythrocyte sedimentation rate (DAS28-ESR) [2,4].

The macrophage migration inhibitory factor (MIF) is a key cytokine in the progression and pathogenesis of RA due to its ability to induce the expression of inflammatory cytokines and molecules that degrade tissue, as well as its capacity to promote proliferation and survival of fibroblast-like synoviocytes, stimulate neutrophil chemotaxis and regulate the process of angiogenesis and osteoclast differentiation [5,6]. These autocrine and paracrine functions exerted by MIF are mediated by its canonical and non-canonical receptors/receptors [7]. The ‘‘canonical’’ receptor is a type II transmembrane protein that consists of a single helix and requires binding to a co-receptor to perform signal transduction (CD74/CD44), while the ‘‘non-canonical receptors” are G-protein-coupled receptors for CXCR2, 4, which engage chemokines containing the Glu-Leu-Arg (ELR) motif, and an atypical non-G protein-associated receptor (CXCR7) [8]. More recently D-dopachrome tautomerase (DDT) or MIF2 has been identified as a new analogue of MIF that seems to share most but not all the biological activities of MIF. However, so far DDT has only been shown to bind to activate the canonical pathway via binding to CD74 receptor [7,9].

The first receptor described for MIF was CD74, a membrane receptor also known as the invariant chain of MHC Class II or li. CD74, unlike the non-canonical receptors, does not bind to any other ligand other than MIF and requires the recruitment of the CD44 co-receptor for signal transduction [7,10]. The interaction between MIF and CD74 is designated as canonical. CD44 is a glycoprotein that also mediates cell–cell adhesion and cell–matrix interactions and is involved in cell migration, tumor invasion, metastasis, and angiogenesis [11].

CD74 is also found in the circulation in a membrane-truncated soluble form (sCD74), which forms complexes with circulating MIF and MIF-2, acting as a decoy receptor to negatively regulate MIF signaling. Therefore, sCD74 provides a mechanism for down-regulation of MIF signal transduction and could control the inflammatory effects of this cytokine [12].

On the other hand, CXCR2, CXCR4, and CXCR7 are non-canonical receptors for MIF [7,13,14]. CXCR2 is a receptor for chemokines with ELR + type motifs such as CXCL8 (IL-8) or CXCL1/GRO-α, whose expression is mainly restricted to monocytes and neutrophils. In contrast, in T lymphocytes, CXCR2 expression is practically null [15,16]. CXCR4 is a G-protein-coupled receptor (GPCR) composed of seven transmembrane helices, which is mainly expressed by mononuclear cells and progenitor cells in the bone marrow [17]. CXCR7, also called atypical chemokine receptor 3 (AKCR3), is the most recently described receptor for MIF. This is an atypical receptor that exerts signals through β-arrestin, which functions as a scaffold protein and interacts with various signaling molecules in the cytoplasm in an agonist-dependent manner [8,18,19].

Although the importance of MIF in RA is known, there is a gap regarding the role of its receptors in influencing clinical activity in this disease. Therefore, this study aimed to evaluate the expression of canonical (CD74/CD44) and non-canonical (CXCR2, 4 and 7) MIF receptors in RA patients and establish their association with clinical biomarkers and the disease activity evaluated through DAS28-ESR.

## 2. Materials and Methods

### 2.1. Universe of Study

We recruited a total of 101 RA patients of both genders from the Rheumatology Service of The Hospital Civil de Guadalajara “Fray Antonio Alcalde,” Guadalajara, Jalisco, México. They were classified according to the 2010 American College of Rheumatology (ACR) and the European League Against Rheumatism (EULAR) criteria [20]. The control subjects (CS) group was formed by a total of 9 gender- and age-matched healthy individuals. Patients with other rheumatic, inflammatory and infectious diseases were excluded. The study was carried out taking into account the principles established in the Declaration of Helsinki (2013); the participation of the subjects included in the study was voluntary, with the prior signing of the informed consent letter. The project was approved by the Research, Ethics, and Biosafety Committee of CUCS, Universidad de Guadalajara (CI-04318).

### 2.2. Clinical Assessment

A rheumatologist evaluated all patients and took a record of the clinical and demographic data. Clinical activity was assessed by the DAS28, considering ESR values. According to DAS28 score, patients were stratified in remission (DAS28 < 2.6; *n* = 27), low activity (DAS28 ≥ 2.6 < 3.2; *n* = 16), moderate activity (DAS28 ≥ 3.2 < 5.1; *n* = 35) and high activity (DAS28 ≥ 5.1; *n* = 23). Functional disability was evaluated by the Spanish version of the Health Assessment Questionnaire-Disability Index (HAQ-DI). 

In the RA patients, rheumatoid factor (RF) and high sensitivity C reactive protein (hsCRP) were determined by turbidimetry (A25 Biosystems, Barcelona, Spain). ESR determination was carried out in both study groups by the Wintrobe method. 

### 2.3. Flow Cytometry

The expression of canonical and non-canonical MIF receptors in the membrane of T lymphocytes, B lymphocytes, monocytes, and granulocytes from RA patients and CS was determined by flow cytometry from whole blood samples.

**Figure 1:**
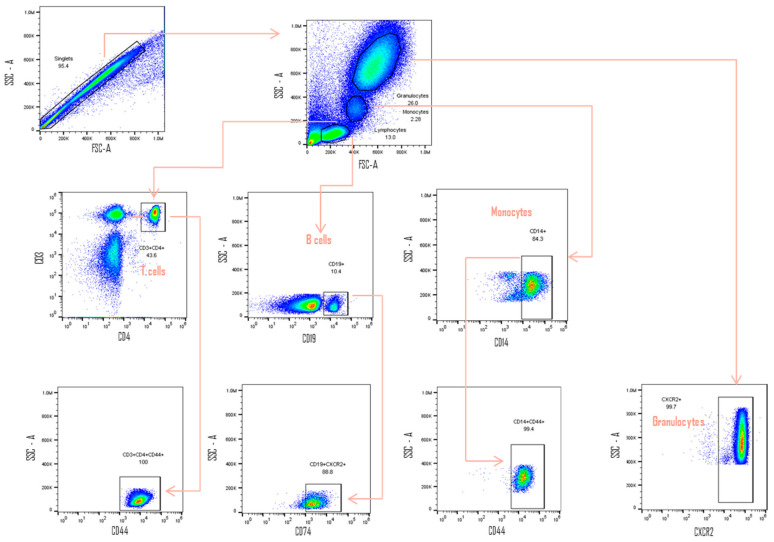
Gating Strategy. Overlapping events were ruled out from the FSC-H/FSC-A analysis. Subsequently, the populations were selected by size and complexity. From the lymphocyte region, T lymphocytes were identified as CD3 + and CD4 + cells, and CD19+ for B lymphocytes. Monocytes were identified as CD14+ cells. Once the three populations had been selected, the positive cells for CD74, CD44, CXCR2, CXCR4 and CXCR7 receptors were identified from the pre-established FMOs (CD44 are represented in CD4+ T lymphocytes, CD74 in B lymphocytes, CD44 in monocytes and CXCR2 in granulocytes). FSC-H/FSC-A; FSC-Height (FSC-H) by FSC-Area (FSC-A). FMO; Fluoresence minus one.

Multicolored labeling was performed using the following corresponding monoclonal antibody panel: CD3 (APC/Cy7 anti-human CD3 Antibody Cat. 300426. Biolegend), CD4 (Alexa Fluor^®^ 488 anti-human CD4 Antibody Cat. 300519. Biolegend), CD14 (PE/Cy7 anti-human CD14 Antibody Cat. 325618. Biolegend), CD19 (PerCP/Cy5.5 anti-human CD19 Antibody Cat. 302230. Biolegend) for the identification of cellular subpopulations. In addition, labeling of CD74 (anti-human PE CD74 Antibody Cat. 326808. Biolegend), CD44 (APC anti-human CD44 Antibody Cat. 338806. Biolegend), CXCR2 (PE anti-human CD182 (CXCR2) Antibody Cat. 320706. Biolegend), CXCR4 (APC anti-human CD184 (CXCR4) Cat. 306510. Biolegend), and CXCR7 (APC anti-human CXCR7 Antibody Cat. 391406. Biolegend) isotype control IgG antibodies conjugated with the same fluorochromes were used as a negative control (Biolegend Inc., San Diego, CA, USA). To accurately identify CD74, CD44, CXCR2, 4 and 7 positive events, the fluorescence minus one experiment was included as an additional negative control. Samples were analyzed in the acoustic focusing Attune^®^ NxT flow cytometer (Life Technologies., Carlsbad, CA, USA). The data obtained were analyzed using FlowJo software v10.0 (TreeStar, Inc., Ashland, OR, USA). First, lymphocytes were gated according to their forward scatter (FC), and side scatter (SS) characteristics. Then, further gates were placed around CD3+ CD4+ T cells, CD19+ B cells, CD14+ monocytes, and granulocytes; subsequently, canonical and non-canonical receptor expression was evaluated. The gating strategy to perform these experiments is shown in Figure 1. 

### 2.4. Quantification of Soluble CD74 Levels

The quantification of sCD74 serum levels in both study groups was performed by the ELISA method using the Human CD74 assay (Sigma Aldrich., St. louis, MO, USA Cat. RAB1351) following the manufacturer’s instructions. The detection limit for sCD74 was 25 ng/mL.

### 2.5. Statistical Analysis

Statistical analysis was carried out using SPSS version 25.0 (IBM SPSS Statics for Windows. Armonk, NY: IBM Corp.) and GraphPad Prism version 7.0 (GraphPad Software, San Diego, CA, USA) software. Variable distribution was determined using the Shapiro–Wilk normality test. Variables non-normally distributed were presented in median and 5–95th centiles. Categorical variables were presented in percentage and absolute frequency. Comparisons between groups were performed using the Mann–Whitney U and Kruskal–Wallis tests.

## 3. Results

### 3.1. Clinical Features of RA Patients

Clinical and demographic characteristics of RA patients are summarized in Table 1. A total of 80.2% of the patients included in the study were women (81/101) and 19.8% were males (20/101). A similar proportion was observed for CS (*n* = 9), with a total of eight women and one man. The median age for the RA patients’ group was 54 years and 51 years for the CS group, and the median of disease evolution was 7 (0.6–40) years.

The median value for the ESR in the patients’ group was 37 mm/h (3–64), while for the hsCRP it was 6 mg/L (0.3–54). According to the clinical records, all of the included patients were seropositive. When determining RF in a total of 76 randomly selected patients, the mean value of the titers was 76 IU/mL; in addition, most of these (67.1%) presented high positive titers (≥60 IU/mL). In contrast, the remaining 32.9% showed positive titers (≥20 <60 IU/mL).

RA patients also had mild disability according to the Spanish HAQ-DI score (0.62). Regarding pharmacological treatment, most of the patients were under different treatment schemes with one or more disease-modifying anti-rheumatic drugs (DMARDs) and/or nonsteroidal anti-inflammatory drugs (NSAIDs). 

When stratifying patients by disease activity, it was observed that the ESR and hsCRP values were higher in the groups with the highest disease activity (*p* = 0.000 and *p* = 0.001, respectively). Groups were similar in age, time of disease evolution, and treatment. RA patients with high disease (DAS28 > 5.1) showed higher disability according to the Spanish HAQ-DI score. 

### 3.2. Expression of the Canonical and Non-Canonical Receptors of MIF and Its Relation to Clinical Activity by DAS28-ESR

#### 3.2.1. T Cells 

**Figure 2:**
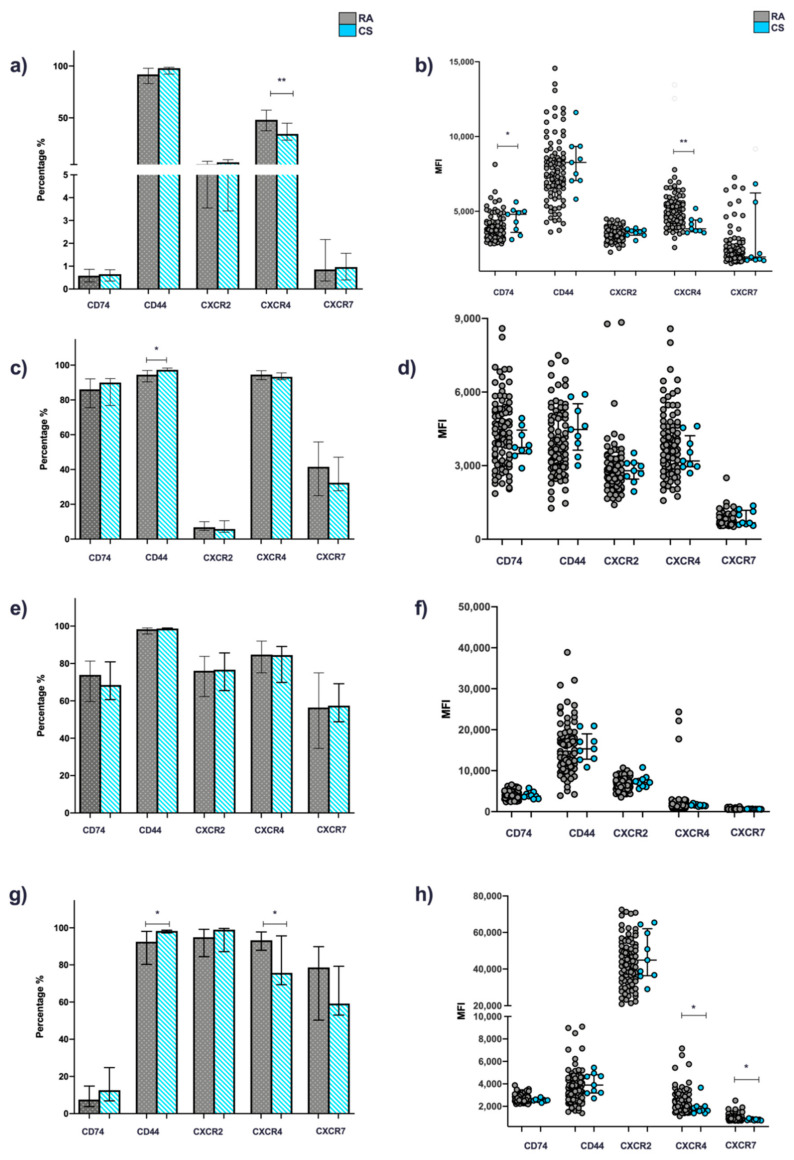
Expression of canonical (CD74/CD44) and non-canonical (CXCR2,4 and 7) MIF receptors in whole blood cells from patients with RA and CS. Percentage and MFI of cells positive for CD74, CD44, CXCR2, CXCR4 and CXCR7. T cells (**a**) and (**b**), B cells (**c**) and (**d**), monocytes (**e**) and (**f**) and granulocytes (**g**) and (**h**) of patients with RA (*n* = 101) vs. SC (*n* = 9). Statistical comparisons between groups were determined using the Mann–Whitney U test (AR vs. SC). * *p* < 0.05; ** *p* < 0.01. RA: rheumatoid arthritis; CS: control subjects; MFI: mean fluorescence intensity.

**Figure 3:**
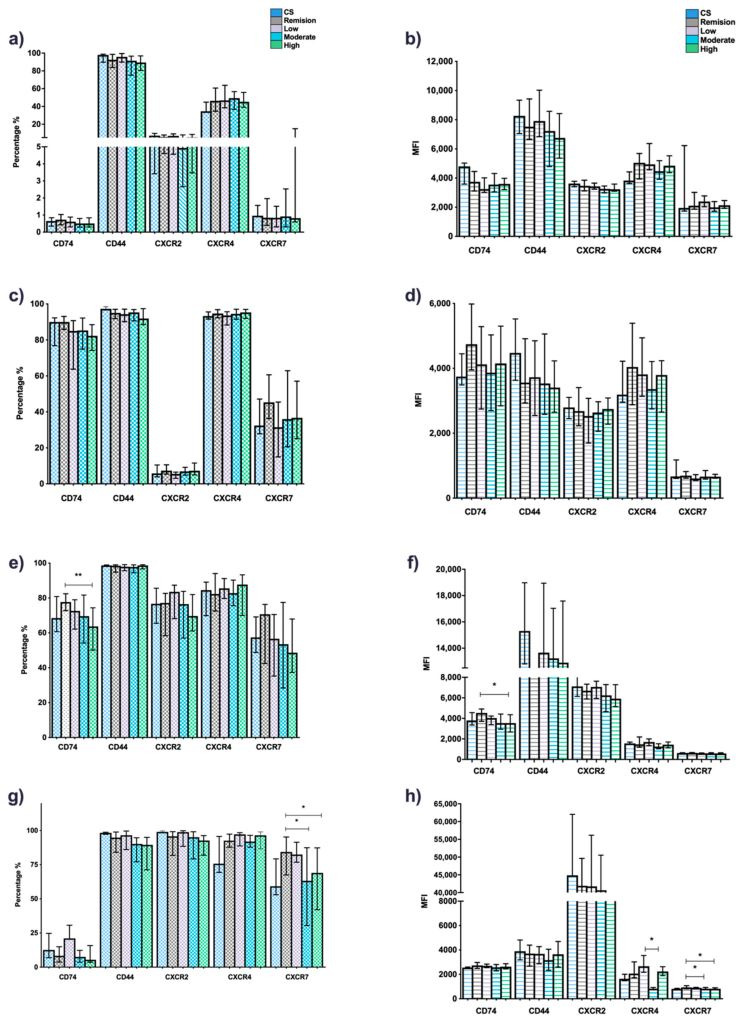
Expression of the canonical (CD74/CD44) and non-canonical (CXCR2, 4 and 7) MIF receptors in whole blood cells from RA patients stratified by DAS28-ESR. Percentage and MFI of cells positive for CD74, CD44, CXCR2, CXCR4 and CXCR7. T cells (**a**) and (**b**), B cells (**c**) and (**d**), monocytes (**e**) and (**f**) and granulocytes (**g**) and (**h**), of patients with RA (*n* = 101) stratified by the DAS28-ESR: remission (<2.6, *n* = 27), low activity (≥2.6 < 3.2, *n* = 16), moderate activity (≥3.2 < 5.1, *n* = 35), high activity (≥5.1, *n* = 23). Data presented in median (p5–p95). Statistical comparisons between groups were determined using the Kruskall–Wallis test (DAS28). * *p* < 0.05; ** *p* < 0.01. RA: rheumatoid arthritis; CS: control subjects; DAS28, disease activity score 28; MFI: mean fluorescence intensity.

When comparing expression patterns of canonical and non-canonical receptors in cells from RA patients and CS, the following was observed: the percentages and MFI of CD44 positive cells were higher in CS than RA patients. This trend was also observed for CD74, CXCR2, and CXCR7; however, only the difference observed for the MFI expression of CD74 was statistically significant (*p* < 0.05). Conversely, for CXCR4, a higher percentage of positive cells was observed, as well as MFI in RA patients in comparison to CS (*p* < 0.05) (Figure 2a,b). No significant difference was observed regarding the comparison between clinical activity groups (Figure 3a,b).

#### 3.2.2. B Cells

In the case of B cells, a higher percentage of positivity and MFI for CD44 was found in CS compared to RA patients (*p* < 0.05 and *p* > 0.05, respectively); similar results were observed for CD74. Contrarily, for CXCR2, CXCR4, and CXCR7, greater expression was observed in RA patients compared to CS; however, these differences were not statistically significant (*p* > 0.05) (Figure 2c,d). 

According to clinical activity, the percentage of CD74+ and CD44+ cells and MFI were higher in the remission group, followed by the low activity group in comparison to the rest of the groups, although these associations showed no significant differences (*p* > 0.05) (Figure 3c,d).

#### 3.2.3. Monocytes and Granulocytes 

For granulocytes (Figure 2g,h), a higher expression (percentage and MFI) of CXCR4 was found in patients with RA compared to CS (*p* < 0.05).

On the other hand, a higher percentage of CD44+ granulocytes were observed in CS compared to RA patients (*p* < 0.05). For monocytes, the expression was constitutive (100%) in both groups (*p* > 0.05). The percentage of CXCR2+ cells and MFI were higher in CS compared to patients for both monocytes and granulocytes (*p* > 0.05). On the other hand, for CXCR7, no significative differences were observed in monocytes, while in granulocytes the percentage of CXCR7+ cells (*p* > 0.05) and MFI (*p* < 0.05) were higher in patients than in the CS group (Figure 2e–h).

Regarding granulocytes, a higher percentage of CD74+ granulocytes, as well as MFI were observed in the CS group compared to RA patients, while in monocytes the opposite was observed (*p* > 0.05) (Figure 2e–h). 

According to clinical activity, monocytes from remission RA patients showed a higher CD74 expression (percentages and MFI, *p* < 0.005) compared to the high clinical activity group. For CXCR7, the same was observed; however, these differences were not significant (*p* > 0.05) (Figure 3e,f).

The percentage of CXCR7+ cells and MFI were increased in granulocytes of patients in remission compared to the moderate and high activity groups (*p* < 0.05). The same trend was observed for the CXCR4 MFI, where patients with low activity had higher expression than those in the moderate activity group (*p* < 0.05) (Figure 3e–h). 

### 3.3. Serum CD74 Levels in Patients with RA and CS

**Figure 4:**
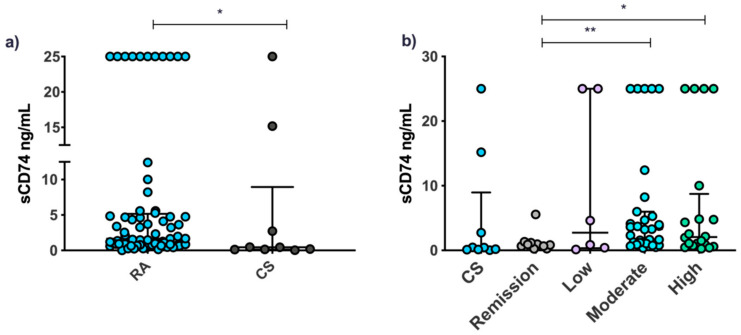
Serum CD74 levels in patients with RA and CS. Serum CD74 levels in patients with RA (*n* = 101) and CS (*n* = 9) (**a**). Serum CD74 levels of RA patients stratified using the DAS28-ESR: remission (<2.6, *n* = 27), low activity (≥2.6 < 3.2, *n* = 16), moderate activity (≥3.2 < 5.1, *n* = 35), high activity (≥5.1, *n* = 23) (**b**). Data presented as median (p5–p95). Statistical comparisons between groups were determined using the Mann–Whitney U (AR vs. CS) and Kruskall–Wallis (DAS28) tests. * *p* < 0.05; ** *p* < 0.01. RA: rheumatoid arthritis; CS: control subjects; DAS28, disease activity score 28.

Serum CD74 levels were quantified in patients with RA (*n* = 71) and CS (*n* = 9). Higher levels were observed in RA patients than CS (*p* < 0.05) (Figure 4a). According to disease activity, evaluated by the DAS28-ESR, patients with moderate and high disease activity had higher levels than those in remission (*p* < 0.01 and *p* < 0.05, respectively) (Figure 4b).

## 4. Discussion

Peripheral blood cells, cytokines and inflammatory molecules play important roles in the perpetuation of the autoimmune process in RA [21]. These may change according to the clinical activity of each patient. Therefore, evaluating the expression of molecules that are associated with the progression and activity of the disease can contribute to a better understanding of the pathological mechanisms involved in the onset and progression of RA to develop therapies that effectively treat patients at each stage of the disease.

Despite what is known about MIF and its important role in the development of RA, to date it has not been described whether there is any association between the expression of its receptors and the different stages of clinical activity in RA. For the reasons stated, the main objective of this study was to evaluate the expression of the canonical (CD74/CD44) and non-canonical (CXCR2, 4 and 7) MIF receptors in patients with RA and to establish their association with the activity of the disease evaluated through the DAS28-ESR.

Regarding MIF canonical receptor complex (CD74/CD44), our study showed that the percentage of CD44+ B cells and granulocytes was greater in CS than RA patients. These results partly agree with those reported by Kelleher et al., where it was described that the expression of CD44 in peripheral blood cells (mainly T cells) is lower compared to the expression in synovial fluid cells, and in turn, CD44 expression in the periphery does not differ significantly between patients with RA and CS [22].

Although the reason for the decrease observed in the present study in CD44+ B cells and granulocytes in patients with RA compared to CS is not known, our findings are possibly related to the fact that these determinations were made in peripheral blood cells, which could not be a reflection of the in situ role of CD44 in RA, where it contributes significantly to cell adhesion and migration as well as in different stages of their activation. Another important aspect to take into account is that the gene that codes for CD44 can go through alternate splicing and, in this way, generate, in theory, up to 800 isoforms. In this context, various studies have reported different functions and expression patterns for these isoforms in a manner dependent on the variant exon they possess [11,23]. Therefore, it is worth delving into these results in subsequent studies to accurately describe the regulatory mechanisms of CD44.

The CD74 MIF receptor had a higher MFI in B cells from CS than patients; in addition, when stratifying by clinical activity groups, a higher percentage of expression and MFI in monocytes was observed in the remission group compared to active patients. This low CD74 expression may be due to the regulatory mechanisms described for this molecule; either β-arrestin-mediated endocytosis after ligand binding as described for Xie et al., 2011, or the generation of a soluble form as a counter-regulation mechanism, where sCD74 could be acting as a decoy receptor that binds and neutralizes circulating MIF in serum [12,24]. This last hypothesis can be supported because we observed that sCD74 levels were higher in RA patients compared with CS and, in turn, in the patients with moderate and high activity compared to those in remission. A plausible possibility is that the disclosed ectodomain shedding of CD74 provides a mechanism for the downregulation of MIF signal transduction and may play a critical role in controlling the proinflammatory actions of this cytokine. Regarding this, in the work of Assis et al., 2014, it was suggested that circulating sCD74 can inhibit MIF-dependent phosphorylation of ERK1/2 in human primary skin fibroblasts, thereby being able to neutralize the inflammatory signaling activity of MIF [25]. Given its possible role as a counter-regulatory factor for the effects of MIF, knowing the molar relationship between sCD74/circulating MIF can be very useful as indicator of inflammatory activity.

Although previous studies reported serum levels of sCD74 in different inflammatory conditions such as biliary cirrhosis, autoimmune hepatitis, and acute lung disease [23,24], the present study, to our knowledge, is the first study to report sCD74 serum levels in RA patients.

Interestingly, when evaluating the expression of CXCR4, a behavior different from that of the rest of the evaluated receptors was observed, with higher expression in all populations in patients with RA compared to CS. The expression of this receptor was higher in turn in the active patients compared to the group in remission. The results agree with those reported by Nagafuchi et al. in 2016 that describe an increase in the expression of CXCR4 in memory CD4 + T cells of RA patients carrying the *HLA-DRB1* genotype [26]. The overexpression observed in peripheral cells of patients compared to CS may also be directly associated with the ability of IL-17A to induce the expression of CXCR4 since elevated serum levels of this cytokine have also been described in patients with RA [27]. In addition to reiterating the role of CXCR4 as an essential component of the inflammatory process, these findings could explain the increase in its concentrations in RA patients compared to CS.

In the case of CXCR7, a behavior similar to that of CXCR4 was observed, with a higher MFI in granulocytes from RA patients. Interestingly, when comparing groups by disease activity, the opposite was observed, with a higher percentage of expression and MFI in the remission group compared to those patients with moderate and high activity. In this context, CXCR7 has been described as a decoy receptor that binds and internalizes CXCL12, thus indirectly modulating the function of CXCR4 by modifying the bioavailability of its ligands [28]. In addition to this, it has been reported that the binding of CXCR7 to its canonical ligands (CXCL12 and CXCL11) does not elicit G-protein coupled second messenger responses but rather is coupled to β-arrestin dependent signaling. CXCR7 is rapidly internalized via a β-arrestin dependent pathway and recycled to the cell surface upon binding to its agonist.

After this internalization, CXCR7 is not degraded; instead, it directs its ligand to lysosomes, which results in its degradation [29].This mechanism has also been described for its binding to non-canonical ligand-MIF, where the MIF:CXCR7 interaction can lead to the internalization of the cell surface of this receptor in a mechanism similar to the internalization caused by CXCL12 [8].

In an integral way, the results obtained for CXCR4 and CXCR7 can be explained because CXCR7 has a high affinity for CXCL12 (10 times more than CXCR4) and acts as a decoy receptor that eliminates CXCL12 from the extracellular environment. Such CXCR7-dependent regulation of local CXCL12 availability ultimately leads to reduced CXCL12/CXCR4 signaling. In this way, CXCR7 can positively regulate the expression of CXCR4 by avoiding endocytosis of this receptor after its exposure to excessive concentrations of CXCL12 [29,30].

The present work provides valuable information about the possible role of the canonical and non-canonical MIF receptors as specific markers of the disease evaluated by the DAS28-ESR. As it has been previously established, these receptors, together with their ligands, actively participate in the pathogenesis of RA; however, various regulatory mechanisms, as well as the state of the inflammatory process, can affect their expression patterns, as evidenced by our results. In addition, as several specific inhibitors of MIF are being advanced from the preclinical setting to human patients our data may suggest the possibility that some of the analytes presently evaluated may represent suitable biomarkers predictive for a therapeutic response to MIF inhibitors in patients with RA and possibly other immunoinflammatory and autoimmune diseases [9].

Among the limitations of this study is the limited number of CS included as well as the flow cytometry panel used, which prevented us from performing the receptor co-expression analysis. These limitations should be considered for later work in our research group.

In later works, it will be necessary to evaluate the expression of the canonical and non-canonical receptors of MIF at the mRNA level to understand the mechanisms that regulate their expression, as well as to quantify the levels of the canonical ligands of CXCR2, 4 and 7, CXCL12 and CXCL8, in serum of RA patients evaluated by DAS28-ESR to precisely know the role of these molecules in the different clinical activity groups. 

## Figures and Tables

**Table 1:** Clinical and demographic features of RA patients.

Variable		RA DAS28				*p* Value
	RA Total (*n* = 101)	Remission (*n* = 27)	Low (*n* = 16)	Moderate (*n* = 35)	High (*n* = 23)	
Demographics						
Gender ^b^						
Female Male	80.2 (81/101) 19.8 (20/101)	59.3 (16/27) 40.7 (11/27)	87.5 (14/16) 12.5 (2/16)	85.7 (30/35) 14.3 (5/35)	91.3 (21/23) 8.7 (2/23)	- -
Age (years) ^c^	54 (19–91)	52 (23–73)	53.5 (40–91)	54 (19–81)	57 (22–88)	0.681
Disease status						
DAS28	3.61 (0.97–7.5)	2.17 (0.97–2.58)	2.95 (2.63–3.17)	4.13 (2.96–5.07)	5.65 (5.17–7.56)	0.000
Disease evolution (years) ^c^	7 (0.6–40)	4.5 (2–25)	7 (1–23)	9 (1–35)	8 (1–40)	0.237
Clinical assessment Rheumatoid Factor (UI/mL) ^c^ Rheumatoid Factor Negative (<20 UI/mL) Positive (≥20 <60 UI/mL) High Positive (≥60 UI/mL) hsCRP (mg/L) ^c^ ESR (mm/h) ^c^ Spanish HAQ-D score ^c^	41.6 (42/101) - 32.9 (25/76) 67.1 (51/76) 6 (0.3–54.5) 37 (3–64) 0.62 (0–2.87)	56 (34.26–89.58) - 50 (9/18) 50 (9/18) 2.1 (0.3–17.2) 18 (3–40) 0.125 (0–0.88)	80.9 (70.34–88.27) - 20 (1/5) 80 (4/5) 7.3 (4.1–47.9) 31.5 (6–53) 0.5 (0–2.38)	79.9 (38.8–89.8) - 33.3 (11/33) 66.7 (22/33) 5.3 (1.2–54.5) 43 (13–55) 0.75 (0–2.63)	73.1 (36.7.24–87.5) - 20 (4/20) 80 (16/20) 10.2 (2.3–42.7) 43 (20–64) 1.4 (0.13–2.87)	0.022 - - - 0.001 0.000 0.000
Treatment ^b^						
NSAIDs	41.6 (42/101)	40.7 (11/27)	56.3 (9/16)	40 (14/35)	34.8 (8/23)	-
Methotrexate	80.2 (81/101)	81.5 (22/27)	81.3 (13/16)	77.1 (27/35)	82.6 (19/23)	-
Methotrexate (mg/week) ^c^	15 (0–60)	15 (0–60)	12.5 (0–15)	15 (0–25)	15 (0–25)	-
Sulfasalazine	80.2 (81/101)	85.2 (23/27)	62.5 (10/16)	77.1 (27/35)	91.3 (21/23)	-
Sulfasalazine (mg/12 h) ^c^	500 (0–3000)	500 (0–1000)	375 (0–2000)	500 (0–3000)	500 (0–3000)	-
Antimalarial	31.7 (32/101)	40.7 (11/27)	37.5 (6/16)	31.4 (11/35)	17.4 (4/23)	-
Antimalarial (mg/12 h) ^c^	0 (0–100)	0 (0–100)	0 (0–75)	0 (0–100)	0 (0–75)	-
No treatment	3 (3/101)	3.7 (1/27)	0 (0/16)	2.9 (1/35)	4.3 (1/23)	-

^b^ Data in percentage (*n*), ^c^ Median data (range), NSAIDs: non-steroidal anti-inflammatory drugs, DAS28: Disease Activity Score 28. HAQ-Di: Health assessment questionnaire disability index, hsCRP: high sensibility C Reactive Protein, ESR: erythrocyte sedimentation rate, RA: rheumatoid arthritis, CS: control subject.
